# Positivity Rate of PD-L1 Expression and Its Clinical Significance in Vulvar Cancer: A Systematic Review and Meta-Analysis

**DOI:** 10.3390/ijms26104594

**Published:** 2025-05-11

**Authors:** Stefanos Flindris, Crysoula Margioula-Siarkou, Christos V. Chalitsios, Georgia Margioula-Siarkou, Emmanouela-Aliki Almperi, Aristarchos Almperis, Effrosyni Styliara, Konstantinos Flindris, Minas Paschopoulos, Iordanis Navrozoglou, Konstantinos K. Tsilidis, Konstantinos Dinas, Stamatios Petousis, Georgios Markozannes

**Affiliations:** 1 2nd Department of Obstetrics and Gynecology, Aristotle University of Thessaloniki, 546 42 Thessaloniki, Greecealemma300@gmail.com (E.-A.A.);; 2 Department of Hygiene and Epidemiology, University of Ioannina, 451 10 Ioannina, Greece; 3 Department of Radiology, University Hospital of Ioannina, 455 00 Ioannina, Greece; 4 Department of Ophthalmology, General Hospital of Ioannina ‘G. Hatzikosta’, 454 45 Ioannina, Greece; 5 Department of Obstetrics and Gynecology, University Hospital of Ioannina, 455 00 Ioannina, Greece; 6 Department of Epidemiology and Biostatistics, School of Public Health, Imperial College London, London SW7 2AZ, UK

**Keywords:** PD-L1, positivity rate, detection rate, vulvar cancer, overall survival, progression-free survival

## Abstract

The prevalence and prognostic value of programmed death ligand 1 (PD-L1) expression, as a potential biomarker in vulvar squamous cell carcinomas (VSCCs), remain underexplored. We searched the PubMed, Scopus, Embase, and Cochrane Library databases until July 2024 for articles examining PD-L1 expression in VSCCs. Random-effects meta-analyses summarized PD-L1 expression overall and in subgroups by immunohistochemistry antibody type, positivity cutoff, tumor stage, and HPV positivity. Additionally, random-effects meta-analyses summarized the association between PD-L1 positivity and cancer prognosis. We included 26 studies comprising 1912 VSCC cases. The summary PD-L1 positivity rate in tumor cells was 59.9% (95% confidence interval [CI]: 47.7–71.4%; I^2^ = 96%, *n* = 26), influenced by the different cutoff thresholds utilized to define PD-L1 positivity. Compared to tumor cells, positivity rates were higher in intratumoral immune cells (75.6%; 95%CI: 52.9–92.5; I^2^ = 95.4%, *n* = 6) and peritumoral cells (78.9%; 95%CI: 54.4–95.5%; I^2^ = 91%, *n* = 3) but with overlapping 95%CIs. No heterogeneity was observed in the rates by tumor stage or HPV status. Positive PD-L1 expression was associated with worse overall (hazard ratio [HR] = 1.43; 95%CI: 1.06–1.93; I^2^ = 28.9%, *n* = 7) and progression-free survival (HR = 1.57; 95%CI: 1.07–2.3; I^2^ = 38.3%, *n* = 5). The PD-L1 expression rate in VSCC tumor cells varied across studies, was influenced by differences in immunohistochemical evaluation, and was identified as an unfavorable prognostic factor. Large, prospective, multicenter studies with standardized protocols are crucial to further elucidate the clinical significance of PD-L1 expression in VSCCs.

## 1. Introduction

Vulvar cancer (VC), accounting for 3% to 5% of all gynecological cancers, ranks fourth in prevalence behind cancers of the uterus, ovaries, and cervix [1,2]. Over 47,000 new cases occurred in 2022, with an age-adjusted incidence rate of 0.8 per 100,000 women [3]. Predominantly composed of squamous cell carcinomas, VC is etiologically classified based on its association with human papillomavirus (HPV), delineating distinct disease profiles [4]. The recent World Health Organization (WHO) classification underscores the importance of confirming HPV status through molecular analysis or p16 immunohistochemistry, acknowledging the dichotomy in morphology between HPV-associated and HPV-negative vulvar squamous cell carcinomas (VSCCs) [5]. While the former primarily affects younger women and is linked to better outcomes, the latter, often associated with lichen sclerosis and TP53 mutations, tends to afflict older women [6,7].

Current therapeutic approaches for VC include surgery, radiation therapy, and systemic chemotherapy, often administered in a multimodal approach depending on the stage of the disease [2,8]. Surgical interventions range from wide local excision to radical vulvectomy with inguinofemoral lymphadenectomy [8,9,10]. Adjuvant therapies are crucial in managing advanced cases and preventing recurrence [11,12]. However, challenges persist in achieving optimal outcomes, with concerns regarding treatment-related morbidities, such as impaired sexual function and lymphedema, impacting the quality of life for survivors [13]. Checkpoint inhibitors targeting the programmed cell death protein 1/programmed cell death ligand 1 (PD-1/PD-L1) pathway emerge as promising therapeutic agents across various malignancies [10,11,14,15,16]. Therapeutic blockade of this pathway through immunotherapeutic interventions holds the potential to impede tumor cells from evading immune surveillance [17]. PD-L1 expression has garnered attention in determining the clinical efficacy of PD-1/PD-L1 inhibitors [18,19,20,21]. Immunohistochemical evaluation of PD-L1 expression in tumor tissue is often quantified using a breadth of immunohistochemical measures, including tumor cells score (TC), tumor proportion score (TPS), immune cell score (ICS), or combined positive score (CPS) [22,23]. Diverse measures and thresholds, as well as approved immunohistochemical assays, are tailored to specific treatment indications within clinical practice [4,24,25].

In VSCCs, the role of this pathway has drawn considerable interest [26,27]. By binding the PD-1 receptor to T cells, activation and differentiation are influenced [28]. Tumor-infiltrating immune cells (ICs) can induce vascular endothelial growth factors, interferons and other cytokines, which upregulate the PD-L1 expression [6,29], affecting various intracellular signaling pathways, transcriptional, and translational levels, and resulting in cancer progression and metastasis [30]. Relevant research on the tumor microenvironment and the role of the PD-1/PD-L1 pathway supports the blockade of this pathway to invert the immune escape and tumor growth in cancer cells [1,21]. Nevertheless, the overall PD-L1 expression rate and its prognostic role in VSCCs are unclear. In this systematic review and meta-analysis we aim to comprehensively assess evidence on the PD-L1 expression in VSCCs, overall, and across multiple subgroups and settings, and also to quantify its association with survival, potentially contributing valuable insights to the current scientific discourse.

## 2. Materials and Methods

We followed the Preferred Reporting Items for Systematic Reviews and Meta-Analyses (PRISMA) statement for preparing this manuscript [31]. This study is registered in PROSPERO (CRD42024556252).

### 2.1. The Search Strategy

We searched the PubMed, Scopus, Embase, and Cochrane Library databases from inception to the 8 July 2024. The following search algorithm was used in PubMed and was translated accordingly for the other databases: (PD-L1 OR Programmed Death-Ligand 1 OR CD274 OR B7-H1) AND (Detection rate OR Expression OR Immunohistochemistry OR IHC OR Biomarker OR Assessment OR Evaluation) AND (Clinical significance OR Prognosis OR Survival OR Overall Survival OR Progression-free Survival OR Outcome OR Predictive value OR Therapeutic implication OR Treatment outcome) AND (Vulvar cancer OR Vulvar carcinoma OR Vulva cancer OR Vulva carcinoma OR Vulvar neoplasms OR Vulva neoplasms OR vulvar squamous cell carcinoma). Reference lists of all articles meeting the inclusion criteria were checked to identify additional potentially eligible studies.

### 2.2. Inclusion and Exclusion Criteria

We included observational studies and clinical trials that assessed (1) the PD-L1 positivity in tumor cells among VSCC cases by immunohistochemistry and (2) the association of PD-L1 expression with clinical outcomes, including overall survival (OS), cancer-specific survival (CSS), disease-free survival (DFS), recurrence-free survival (RFS), and progression-free survival (PFS). The included studies had to report for PD-L1 expression (1) the percentage of positivity rate with a measure of variation or the number of positive cases and the total number of VSCC cases and for the association with clinical outcomes (2) a measure of association (odds ratio [OR], hazard ratio [HR], or relative risk [RR]), along with the 95% confidence interval (CI) or another measure of variation. We only included papers written in English.

### 2.3. Study Selection, Data Extraction, and Risk of Bias

Four reviewers (SF, SP, MSC and CVC) independently assessed titles, abstracts, and the full text of articles, and consensus on eligibility was reached by discussion. Two reviewers (SF and GM) independently extracted data from the studies, including study information (e.g., the name of the lead author and publication year), population characteristics (e.g., country, number of participants, sample size, disease stage, antibody clones used, immunohistochemical staining evaluations, PD-L1 positivity rates, cutoff thresholds, HPV status, and the prognostic value of reported PD-L1 expression), and study results (e.g., effect estimates and 95%CI or variation measure from the maximally adjusted multivariable models, if reported). If only univariable (unadjusted) estimates were reported, they were retained to avoid loss of information. A third reviewer (CCV) resolved any discrepancies. Study quality was assessed using the Newcastle–Ottawa scale (NOS) [32] by two reviewers (SF and GM).

### 2.4. Statistical Analysis

Random-effects meta-analysis models were used to calculate (1) the summary proportion for the PD-L1 positivity and (2) summary estimates of the association of PD-L1 positivity and clinical outcomes. Of note, due to the limited data, DFS, RFS, and PFS were meta-analyzed together under PFS. The I^2^ statistic was used to quantify the percentage of variation across studies due to heterogeneity, with values indicating low (<25%), moderate (25–50%), high (50–75%), and extreme (>75%) variation across studies due to heterogeneity. Subgroup analyses aimed to explore the PD-L1 positivity rate across various clinicopathological characteristics (type of immunohistochemistry antibody assessed, cutoff used to define positivity, tumor stage, and HPV positivity). We performed a leave-one-out sensitivity analysis to evaluate the impact of single studies on the summary estimate. Indication of small study effects bias was based on visual inspection of the funnel plots and Egger’s regression asymmetry test when at least ten studies were included in a meta-analysis. Meta-regression analysis was conducted to investigate the potential impact of sample size on the results. Statistical significance was set at α = 0.05, except for Egger’s regression test, for which α = 0.1 was used, as the test is acknowledged to have low power. Analyses were performed in Stata version 16.1 (StataCorp. College Station, Texas, USA).

## 3. Results

### 3.1. A Description of the Included Studies

Our search yielded 143 records. A total of 38 articles were potentially eligible for full-text screening, of which 26 published studies [1,4,5,7,9,10,17,19,20,24,26,29,33,34,35,36,37,38,39,40,41,42,43,44,45,46] were finally included in our review (Figure S1). Details of the 12 excluded studies during the full-text screening are provided in Table S1. The sample size of the included studies ranged from 18 to 427, encompassing a total of 1912 women with VSCCs aged between 20 and 96 years (Table 1). Most studies were conducted in the USA (*n* = 15, 58%) and Germany (*n* = 3, 12%), two were international studies, and one each was conducted in China, Denmark, Italy, Poland, Portugal, and Switzerland. Most studies included VSCCs of any stage (*n* = 11, 42%), 8 (31%) included non-metastatic VSCCs, two included only advanced/metastatic VSCCs, while five studies did not report cancer stage. All included studies retrospectively assessed the expression of PD-L1 in VSCC tumor cells. The most frequently assessed PD-L1 antibodies were clone 22C3 (*n* = 13, 50%) and SP142 (*n* = 6, 23%), followed by clone E1L3N (*n* = 2), clone SP263 (*n* = 2), clone 28-8 (*n* = 1), CD274 (*n* = 1), and clone 9A11 (*n* = 1). Eight studies examined the association between PD-L1 expression and clinical outcomes, including OS (*n* = 7), PFS (*n* = 5), and CSS (*n* = 1).

### 3.2. PD-L1 Expression in VSCCs

**Figure 1:**
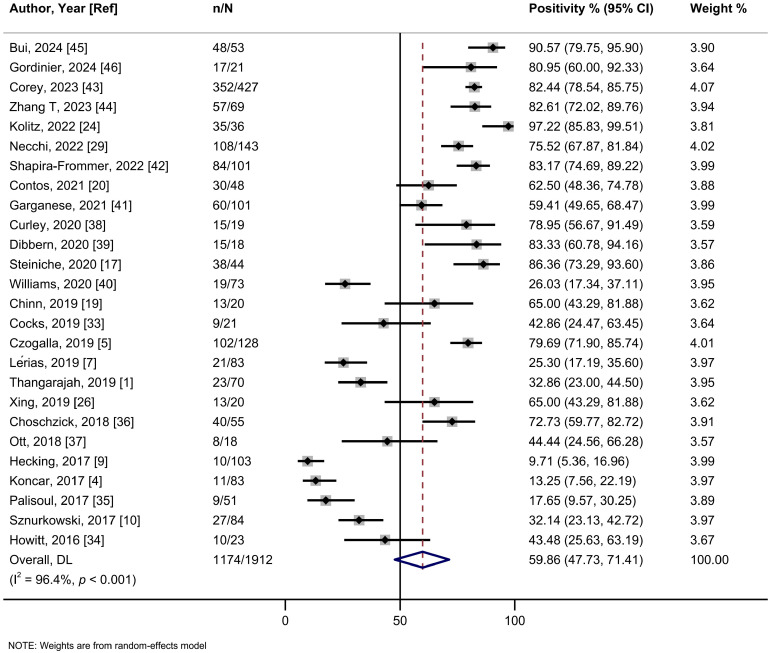
A forest plot of the summary positivity rate of programmed cell death ligand 1 (PD-L1) expression in vulvar cancer. Abbreviations: CI: confidence interval.

The main metrics used to define PD-L1 positivity were tumor proportional score (TPS) and combined proportion score (CPS), while one study employed H-score. Specifically, to define positivity, 13 studies used tumor cells (TC) score. These included 9 studies using TC ≥ 1%, 2 studies using TC ≥ 5%, and 2 studies that defined positivity as TCS ≥ 2% in TC ≥ 5%. Furthermore, 10 studies employed a CPS of ≥1. The summary estimate of PD-L1 positivity rate in VSCCs was 59.9% (95%CI: 47.8–71.4%; I^2^ = 96.4%, 26 studies) (Figure 1, Table 2). The leave-one-out sensitivity analysis did not show any significant influence of single studies on the summary estimate (Figure S2). No asymmetry was detected by visual inspection of the funnel plot or by Egger’s regression test (*p* = 0.36) (Figure S3). A meta-regression analysis did not show a statistically significant association between sample size and positivity rate (*p* = 0.68) (Figure S4).

**Figure 2:**
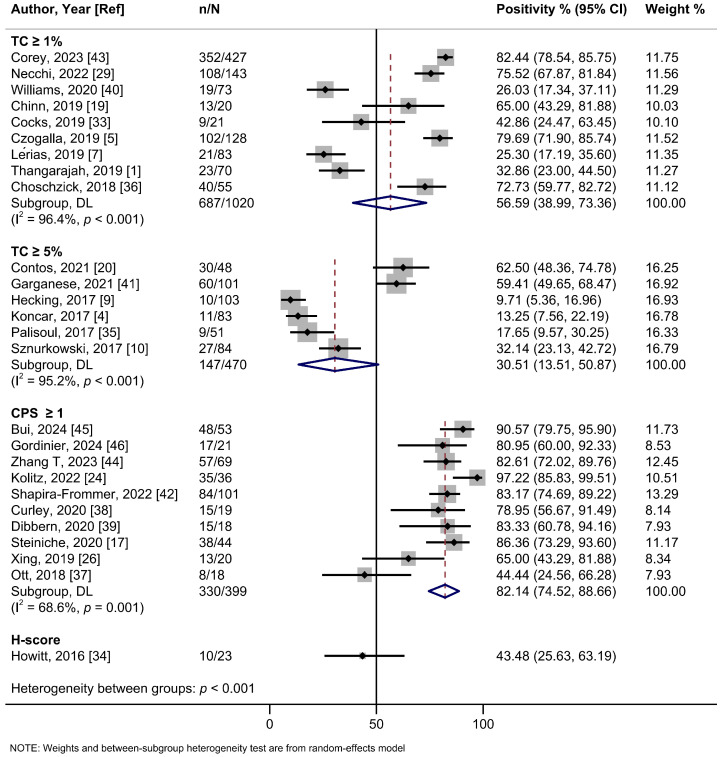
A forest plot of the summary positivity rate of programmed cell death ligand 1 (PD-L1) expression in vulvar cancer, by cutoff thresholds used to assess the expression of PD-L1. Abbreviations: CI: confidence interval.

**Figure 3:**
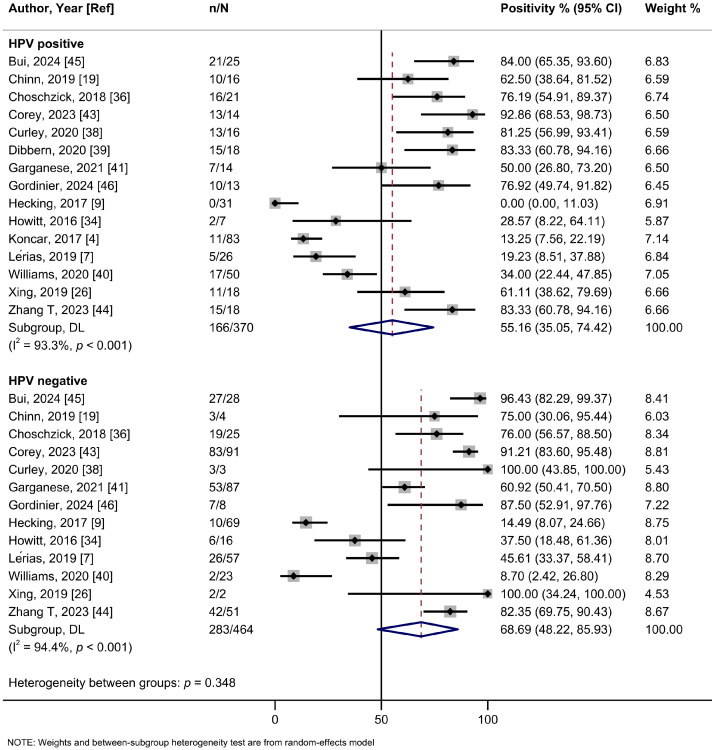
A forest plot of the summary positivity rate of programmed cell death ligand 1 (PD-L1) expression in vulvar cancer, by HPV status (positive and negative). Abbreviations: CI: confidence interval.

**Figure 4:**
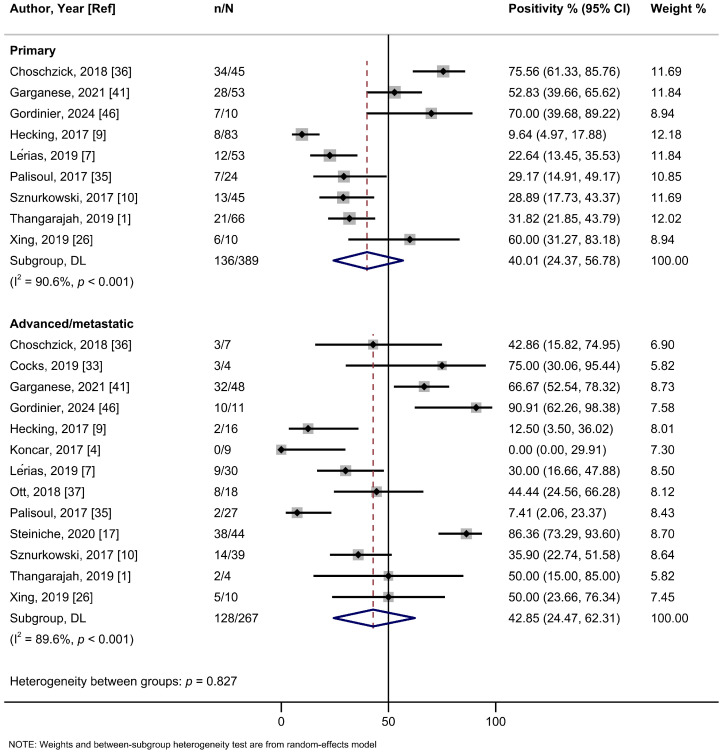
A forest plot of the summary positivity rate of programmed cell death ligand 1 (PD-L1) expression in vulvar cancer, by tumor stage (primary and advanced/metastatic). Abbreviations: CI: confidence interval.

In the subgroup analysis based on the cutoff thresholds used to assess the expression of PD-L1, studies employing a CPS ≥ 1 threshold reported a higher summary estimate of 82.1% (95%Cl: 74.5–88.7%; I^2^ = 68.6%; 10 studies) than those using TC ≥ 1% (56.6%, 95%Cl: 39.0–73.4%; I^2^ = 96.4%; 9 studies), TC ≥ 5% (30.5%, 95%Cl: 13.5–50.9%; I^2^ = 95.2%; 6 studies), or H-score (43.5%, 95%Cl: 25.6–63.2%; one study) (Figure 2, Table 2). Meta-analyses by PD-L1 antibodies were possible for 22C3 (56.9%, 95%Cl: 37.5–75.3%; I^2^ = 96.9%; 13 studies), SP142 (64.3%, 95%Cl: 34.6–89.0%; I^2^ = 97.0%; 6 studies), E1L3N (59.4%, 95%CI: 29.9–85.5; I^2^ = 82.6%, 2 studies), and SP263 (70.2%, 95%Cl: 48.9–87.6%; I^2^ = 91.1%; 2 studies) clones, yielding relatively similar estimates (Figure S5, Table 2). Only one study each assessed clones 28-8, 9A11, and CD274. No evidence of subgroup heterogeneity (*p* = 0.348) was observed for PD-L1 positivity rates among HPV-positive tumors (55.2% (95%Cl: 35.1–74.4%; I^2^ = 93.3%; 13 studies) compared to HPV-negative tumors (68.7%; 95%Cl: 48.2–85.9%; I^2^ = 94.4%; 15 studies) (Figure 3, Table 2). Similarly, no subgroup heterogeneity (*p* = 0.827) was observed for PD-L1 expression across VSCCs of primary stages (40.1%; 95%Cl: 24.4–56.8%; I^2^ = 90.6%; 9 studies) compared to advanced/metastatic stages (42.9%; 95%Cl: 24.5–62.3%; I^2^ = 89.6%; 13 studies) (Figure 4, Table 2).

Further analysis of PD-L1 expression in ICs in VSCCs showed a summary positivity rate of 75.6% (95%CI 52.9–92.5; I^2^ = 95.4%; 6 studies) (Figure S6, Table 2). Limited studies examined the use of different PD-L1 antibodies for ICs. Based on two studies for the 22C3 clone, the summary estimate was 73.1% (95%CI: 8.2–100%; I^2^ = 98.2%) (Figure S7, Table 2). In a subgroup analysis by the cutoff used to assess PD-L1 expression in ICs within VSCCs, studies employing a CPS ≥ 1 threshold reported numerically higher summary estimate of 91.9% (95%CI: 75.8–99.6%; I^2^ = 78.3%; 2 studies), compared to studies using TC ≥1% (74.2 %; 95%CI: 46.0–94.4%; I^2^ = 93.7%; 3 studies), but with overlapping 95%CIs (Figure S8, Table 2). Finally, PD-L1 expression in peritumoral ICs in VSCCs was reported in three studies, with a summary estimate of 78.9% (95%CI 54.4–95.5%; I^2^ = 91%) (Figure S9, Table 2).

### 3.3. PD-L1 Expression and VSCC Prognosis

**Figure 5:**
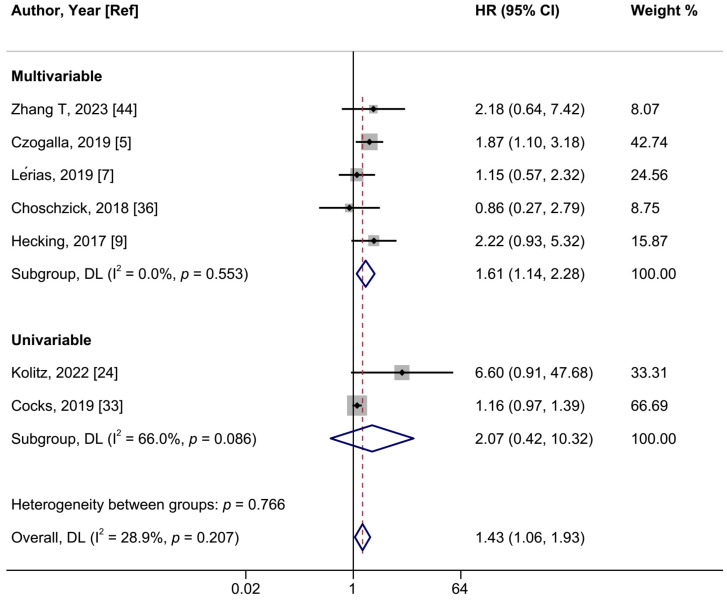
A forest plot of the association between positive vs. negative programmed cell death ligand 1 (PD-L1) expression in intratumoral cells and overall survival in vulvar cancer, stratified by univariable and multivariable-adjusted models. Abbreviations: CI: confidence interval; HR: hazard ratio.

**Figure 6:**
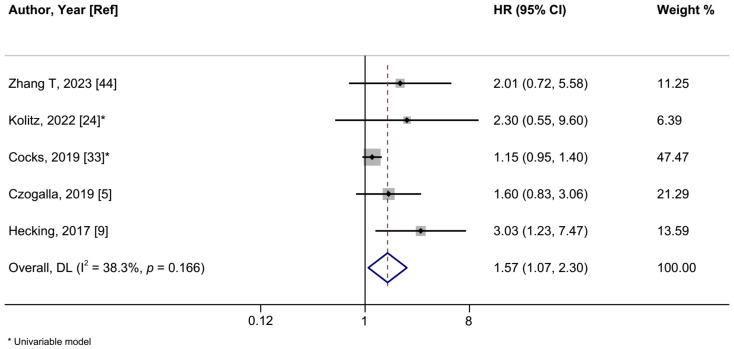
A forest plot of the association between positive vs. negative programmed cell death ligand 1 (PD-L1) expression and vulvar cancer progression-free survival. Abbreviations: CI: confidence interval; HR: hazard ratio.

In a meta-analysis of seven studies, positive PD-L1 expression was associated with a shorter OS in women with VSCCs with a summary HR of 1.43 (95%CI: 1.06–1.93; I^2^ = 28.9%) (Figure 5, Table 2). When we restricted the analysis to the five studies employing multivariable models, the HR was 1.61 (95%CI: 1.11–2.28; I^2^ = 0%). The summary HR of the association between PD-L1 expression and PFS was 1.57 (95%CI: 1.07–2.30; I^2^ = 38.8%; 5 studies) (Figure 6). Cocks et al. was the only study of PD-L1 positivity in tumor cells and CSS, reporting an HR of 1.12 (95%CI: 0.90–1.40) (Table 2) [33]. Four studies examined the association of intratumoral ICs PD-L1 positivity with OS, with a summary HR of 1.44 (95%CI 0.59–3.51; I^2^ = 81.7%) (Figure S10, Table 2). Furthermore, intratumoral ICs PD-L1 positivity was not associated with PFS based on three studies (HR:1.43; 95%CI 0.72–2.84; I^2^ = 43.6%) (Figure S11, Table 2).

The scores for the studies providing association estimates with clinical outcomes ranged from 5 to 9, with 3 receiving a score of 5 or 6 (fair quality) and 5 receiving a score of 7 to 9 (high quality). In summary, the studies with lower scores scored poorly in domains primarily related to adequacy of follow-up of cohorts and for outcomes to occur, and comparability for not appropriately adjusting for confounders (univariable analyses or not adjusting for at least age and grade/stage) (Table S2). When these studies were excluded from a sensitivity meta-analysis on OS (Figure S12), similar results were observed.

## 4. Discussion

This is a systematic review and meta-analysis on PD-L1 expression in tumor, immune, and peritumoral cells as well as its association with clinical outcomes in women with VSCCs. The summary estimate of PD-L1 positivity rate in VSCC tumor cells was 55.5%, exhibiting significant heterogeneity influenced by the assessment method. However, despite exhibiting numerical differences, we did not observe significant heterogeneity of positivity rates depending on the antibodies used, disease stage, or HPV status. In the limited studies assessing PD-L1 in intratumoral immune cells and peritumoral cells, positivity rates were numerically higher compared to tumor cells. Positive PD-L1 expression was associated with worse OS and PFS.

Meta-analyses across various cancer types have shown varied PD-L1 expression rates. For instance, the summary estimates of PD-L1 expression have been reported to be 34.3% in tumor cells and 51.4% in ICs in endometrial cancer [47], 18.7% in tumor cells and 51.2% in ICs in breast cancer [48], and 26% in tumor cells in small-cell lung cancer [15]. Fakri et al. reported 34.4% PD-L1 expression in ICs in non-small-cell lung cancer [49], while Fu et al. found expression rates of 58.1% in cervical cancer tumor cells, 33.8% in endometrial cancer tumor cells, and 37.5% in ovarian cancer tumor cells [50]. In gastro-esophageal cancer, PD-L1 expression ranged from 14% to 24% in tumor cells and reached 35% in ICs [51]. In our study, we observed higher PD-L1 expression in VSCCs, with 86.2% in ICs and 82.4% in peritumoral cells within the tumor microenvironment, suggesting that the PD-1/PD-L1 pathway may be more crucial in VSCC carcinogenesis compared to other cancer sites [47,48,52,53,54]. Therefore, PD-L1 expression in ICs is a key indicator of innate immunity and immunosuppression within the tumor microenvironment. PD-L1 expression on tumor cells has been widely implicated in immune escape and generally results in a poorer prognosis. Meta-analyses on lymphomas [55] and on solid tumors [56] showed that high expression of tumor-cell PD-L1 is associated with lower overall survival. On the other hand, PD-L1 positivity on tumor-infiltrating immune cells, which may reflect a pre-existing anti-tumor immune response, has been associated with better prognosis of other cancer sites, including intrahepatic cholangiocarcinoma [57], and head and neck [58] cancers. Overall, PD-L1 on immune cells may serve as a biomarker of an inflamed, T-cell-rich microenvironment primed for checkpoint blockade, whereas tumor-cell PD-L1 may denote adaptive immune resistance.

Subgroup analysis showed that evaluation cutoff thresholds significantly influenced the summary estimates. Studies using CPS reported higher PD-L1 expression than those using TC. Therefore, it is crucial to examine PD-L1 expression in the tumor microenvironment, not just in tumor cells alone. Our meta-analysis demonstrated that the combined positive score (CPS) yields a significantly higher PD-L1 detection rate in vulvar cancer compared to methods that rely solely on tumor cell evaluation (such as TPS or TC scoring). This result is in line with findings from studies in small-cell lung and triple-negative breast cancers, where CPS by integrating PD-L1 expression on both tumor and immune cells captures a broader spectrum of PD-L1 positivity [15,59]. Furthermore, interassay and interobserver comparability studies have shown that while TPS offers excellent reproducibility, CPS provides enhanced sensitivity by accounting for the immune microenvironment, making it a potentially more clinically relevant cutoff threshold for selecting patients likely to benefit from PD-1/PD-L1-targeted therapies [59,60,61]. The primary source of heterogeneity in our results stemmed from variations in assessment methods and cutoff thresholds across studies. Notably, high heterogeneity persisted even within those subgroup analyses, suggesting additional underlying contributing factors.

In our meta-analysis, VSCC stage was not associated with PD-L1 expression in tumor cells. These findings are contradictory to other meta-analyses in colorectal, pancreatic, endometrial and breast cancer [16,47,54] that reported differences across stages. The activation of the Janus Kinase 2/signal transducers and activators of the transcription 1 (JAK2/STAT1) signaling pathway promotes the overexpression of PD-L1 and tumor progression [40,54]. Other crucial pathways in which PD-L1 interferes are phosphoinositide 3-kinase/protein kinase B (PI3K/Akt) and rat sarcoma/mitogen-activated protein kinase/extracellular signal-regulated kinase (Ras/MEK/Erk) binding to PD-1 reduce TCR-mediated signaling, thereby disrupting them [25,30,35]. The expression of PD-L1 in the tumor cells facilitates immunosuppression, both of which contribute to tumor progression and metastasis [25,38]. While stratification by antibody type, disease stage, or HPV status did not reveal significant differences, these variables may still contribute to the residual heterogeneity. Furthermore, the generally small sample sizes of the included studies that potentially lead to less stable estimates could amplify this variability.

Our results indicate that PD-L1 expression in tumor cells, but not in ICs, is associated with worse OS and PFS among VSCC patients. Despite these findings, our findings suggest that scores evaluating the PD-L1 expression in tumor cells may provide more robust evidence for the treatment and prognosis of VSCCs. Meta-analyses on different cancer sites have shown that PD-L1 is a biomarker of poor prognosis in cervical, breast, endometrial, pancreatic, and colorectal cancer, but not in ovarian cancer [14,16,47,49,50,62,63,64]. Previous research has shown that, in addition to tissue PD-L1, circulating forms such as exosomal PD-L1 and soluble PD-L1 also exist [47,54]. However, further studies are needed to confirm the mechanisms underlying this hypothesis. Understanding the mechanisms of action of the PD-1/PD-L1 pathway is important in elucidating its role in carcinogenesis [53]. Tumor progression is triggered by the binding of PD-L1 to its various receptors via immune escape [65]. However, PD-L1 exerts non-immune proliferative effects on various cancer types [25,27,66]. The expression of PD-L1 both in tumor and ICs could potentially predict responsiveness to targeted therapies [53,54]. It is of paramount importance to cast more light on the research field to better understand the role of this pathway in VSCCs. PD-1/PD-L1 inhibitors such as pembrolizumab, nivolumab, atezolizumab, durvalumab, and avelumab have presented anti-cancer effects on gynecological cancers and cancers originating from other organs [21,52,59,67]. Additionally, recent data indicate that cytotoxic drugs and targeted therapies can influence immune responses. The controversial results of KEYNOTE-028 and KEYNOTE-158, which despite the PD-L1 expression in tumor cells, the objective response rate was higher in PD-L1-negative tumors, indicate that the tumor microenvironment is crucial for the PD-1/PD-L1 pathway for VSCCs [37,42]. Schwab et al. conducted a meta-analysis of those trials, concluding that approximately one-third of women with advanced or recurrent VSCCs might benefit from pembrolizumab treatment, independent of their PD-L1 status, despite shortening of progression-free and OS at 12 months [67]. CheckMate 358 Trial suggests that the treatment with nivolumab in VSCCs is promising and needs further investigation [12].

In subsequent research, it will be vital to standardize PD-L1 testing, using uniform antibodies, scoring methods and cutoffs to minimize variability. At the same time, translational studies should explore high-dimensional immune profiling to uncover co-regulated checkpoints, nanoparticle-mediated siRNA or small-molecule delivery to modulate PD-L1, and CRISPR/Cas genome-editing approaches to knock down or edit PD-L1. These strategies can elucidate PD-L1′s value as a predictive marker and potentially contribute to personalized, combinatorial immunotherapies in vulvar cancer.

Our study has several strengths. While we cannot guarantee that all relevant studies were identified, our thorough literature search across multiple databases with well-defined search terms, coupled with the non-significant results of the small study effects test, suggests that our findings are not affected by publication bias. While substantial heterogeneity was observed, all the included studies utilized samples obtained from surgical excisions of primary tumors, with some also incorporating advanced tumors. The reliance on surgical specimens likely reduces bias, as it facilitates a comprehensive evaluation of PD-L1 expression across diverse disease scenarios [15,47]. Additionally, the use of surgically resected tissues minimizes sampling errors in studies assessing PD-L1 expression in both immune cells (ICs) and tumor cells through appropriate scoring systems [7,10,24,33]. Furthermore, to investigate possible sources of heterogeneity, we conducted extensive subgroup and sensitivity analyses, enabling a thorough assessment of PD-L1 expression across various contexts. Finally, by synthesizing evidence on the prognostic significance of PD-L1 expression and its association with survival outcomes, our study offers valuable insights into the role of the PD-L1 pathway in VSCCs. These findings hold promise for informing future clinical and therapeutic developments.

Despite its strengths, our study also has several limitations that should be acknowledged. Most studies are retrospective and provide relatively small sample sizes. Analytical factors present significant variations, such as different assays for PD-L1 expression, diverse scoring systems (e.g., TC, CPS), disparate cutoff values and various primary antibodies and staining patterns to determine PD-L1 positivity. Some authors propose using quantitative polymerase chain reaction (qPCR) for PD-L1 positivity and analysis to improve validation and comparability across studies [18,47]. Despite performing subgroup and meta-regression analyses, unidentified factors would contribute to the significant heterogeneity, including inherent disparities between the studies beyond random chance, including study design, patients’ characteristics, possible treatment effects, and other factors. Notably, only a small number of studies from the USA and Europe provided data on prognosis, limiting the generalizability of our results. Furthermore, survival data for PD-L1 expression in VSCCs, either in tumor or ICs, are significantly limited. Additionally, different treatment protocols may influence survival outcomes; however, studies usually did not report relevant information. Hence, the estimates reported in our study should be interpreted cautiously.

## 5. Conclusions

In conclusion, we observed that the PD-L1 expression rate in VSCCs was higher than in other gynecological malignancies, either in TCs or ICs, but varied substantially between studies influenced mainly by cutoff thresholds and assessment methods used to assess the expression. Although our study provides preliminary insights into the association of PD-L1 expression in tumor cells with worse VSCC PFS and OS, its reliability as a prognostic biomarker requires further research. Larger prospective studies that comprehensively assess the PD-L1 expression rate using standardized procedures in diverse populations and clinical settings are essential to assess the clinical value of PD-L1 expression and its prognostic implications on VSCCs.

## Figures and Tables

**Table 1:** Characteristics of the included studies.

Author, Year	Country	Mean Age (Range), Years	Sample Size	Stage	Histology and Molecular Status	Clone Antibody	Cutoff	Clinical Outcome
Howitt, 2016 [34]	USA	69 (49–93)	23	NR	VSCC according to p16 status	9A11	H-score	NR
Hecking, 2017 [9]	Germany	64 (26–93)	103	Primary, advanced, metastatic	VSCC according to p16/HPV DNA status	22C3	TCS ≥ 2% in TC ≥ 5%	OS, RFS
Koncar, 2017 [4]	USA	61 (29–85)	83	Primary, advanced, metastatic	VSCC, p53 wild-type	SP142	TC ≥ 5%	NR
Palisoul, 2017 [35]	USA	65 (NR)	51	Primary, advanced, metastatic	VSCC (85%) and AC (15%)	22C3	TCS ≥ 2% in TC ≥ 5%	NR
Sznurkowski, 2017 [10]	Poland	68 (36–85)	84	Primary, advanced	VSCC	22C3	TC ≥ 5%	OS
Choschzick, 2018 [36]	Switzerland	69 (37–89)	55	Primary, advanced, metastatic	VSCC according to HPV RNA status	E1L3N	TC ≥ 1%	OS
Ott, 2018 [37]	Worldwide	59 (18–87)	18	Metastatic	VSCC	22C3	CPS ≥ 1	NR
Chinn, 2019 [19]	USA	NR	20	NR	VSCC according to p16 status	SP142	TC ≥ 1%	NR
Cocks, 2019 [33]	USA	58 (25–87)	21	Primary, advanced	VSCC	E1L3N	TC ≥ 1%	OS, DFS, CSS
Czogalla, 2019 [5]	Germany	71 (20–96)	128	Primary, advanced, metastatic	VSCC	SP263	TC ≥ 1%	OS, PFS
Lérias, 2019 [7]	Portugal	74 (26–93)	83	Primary, advanced	VSCC according to p16/HPV DNA status	22C3	TC ≥ 1%	OS
Thangarajah, 2019 [1]	Germany	62 (48–71)	70	Primary, advanced	VSCC	28-8	TC ≥ 1%	NR
Xing, 2019 [26]	USA	NA (25–79)	20	Primary, advanced, metastatic	VSCC according to p16/HPV RNA status	22C3	CPS ≥ 1	NR
Curley, 2020 [38]	USA	NR	19	NR	VSCC HPV-associated vs. unassociated status	SP142	CPS ≥ 1	NR
Dibbern, 2020 [39]	USA	NR	18	NR	VSCC HPV-associated	SP142	CPS ≥ 1	NR
Steiniche, 2020 [17]	Denmark	68 (66–73)	44	Advanced, metastatic	VSCC	22C3	CPS ≥ 1	NR
Williams, 2020 [40]	USA	62 (25–92)	73	Primary, advanced	VSCC	22C3	TC ≥ 1%	NR
Contos, 2021 [20]	USA	NR	48	NR	VSCC	SP142	TC ≥ 5%	NR
Garganese, 2021 [41]	Italy	78 (48–96)	101	Primary, advanced	VSCC according to p16 status	SP263	TC ≥ 5%	NR
Kolitz, 2022 [24]	USA	60 (NR)	36	Primary, advanced	VSCC	22C3	CPS ≥ 1	OS, PFS
Necchi, 2022 [29]	USA	64 (29–89)	143	Primary, advanced, metastatic	VSCC	22C3	TC ≥ 1%	NR
Shapira-Frommer, 2022 [42]	Worldwide	64 (31–87)	101	Primary, advanced, metastatic	VSCC	22C3	CPS ≥ 1	NR
Corey, 2023 [43]	USA	66 (30–90)	427	Primary, advanced, metastatic	VSCC	SP142	TC ≥ 1%	NR
Zhang T, 2023 [44]	China	67 (29–91)	69	Primary, advanced	VSCC according to p16/HPV IHC status	22C3	CPS ≥ 1	OS, PFS
Bui, 2024 [45]	USA	70.6 (34–96)	53	Primary, advanced, metastatic	VSCC according to p16 status	CD274	CPS ≥ 1	NR
Gordinier, 2024 [46]	USA	64.7 (36–93)	21	Primary, advanced, metastatic	VSCC	22C3	CPS ≥ 1	NR

Abbreviations: CPS: combined positive score; CSS: cancer-specific survival; DFS: disease-free survival; HPV: human papillomavirus; IHC: immunohistochemistry; NR: not reported; OS: overall survival; PFS: progression-free survival; RFS: recurrence-free survival; TC: tumor cells; TCS: tumor cell score; VSCC: vulvar squamous cell carcinoma.

**Table 2:** Summary of results for the PD-L1 expression rate and its association with cancer prognosis in VSCCs.

PD-L1 Expression in Tumor Cells	Positivity	95%CI	I^2^	Studies
**Overall**	59.9%	47.8% to 71.4%	96.4%	26
**By cutoff thresholds**				
CPS ≥ 1	82.1%	74.5% to 88.7%	68.6%	10
TC ≥ 1%	56.6%	39.0% to 73.4%	96.4%	9
TC ≥ 5%	30.5%	13.5% to 50.9%	95.2%	6
H-score	43.5%	25.6% to 63.2%	-	1
**By PD-L1 antibodies**				
E1L3N	59.4%	29.9% to 85.5%	82.6%	2
SP263	70.2%	48.9% to 87.6%	91.1%	2
22C3	56.9%	37.5% to 75.3%	96.9%	13
SP142	64.3%	34.6% to 89.0%	97.0%	6
28-8	32.9%	23.0% to 44.5%	-	1
9A11	43.5%	25.6% to 63.2%	-	1
CD274	90.6%	79.6% to 95.9%	-	1
**By HPV positivity**				
HPV-positive	55.2%	35.1% to 74.4%	93.3%	13
HPV-negative	68.7%	48.2% to 85.9%	94.4%	15
**By cancer stage**				
Primary stages	40.1%	24.4% to 56.8%	90.6%	9
Advanced/metastatic stages	42.9%	24.5% to 62.3%	89.6%	13
**PD-L1 expression in intratumoral immune cells**	**Positivity**	**95%CI**	**I^2^**	**Studies**
**Overall**	75.6%	52.9% to 92.5%	95.4%	6
**By cutoff thresholds**				
CPS ≥ 1	91.9%	75.8% to 99.6%	78.3%	2
TC ≥ 5%	36.9%	27.4% to 47.6%	-	1
TC ≥ 1%	74.2%	46.0% to 94.4%	93.7%	3
**By PD-L1 antibodies**				
CD274	84.9%	73.0% to 92.3%	-	1
E1L3N	66.7%	45.4% to 82.8%	-	1
SP263	91.4%	85.3% to 95.1%	-	1
22C3	73.1%	8.2% to 100%	98.2%	2
28-8	58.6%	46.9% to 69.4%	-	1
**PD-L1 expression in peritumoral immune cells**	**Positivity**	**95%CI**	**I^2^**	**Studies**
**Overall**	78.9%	54.4% to 95.5%	91%	3
**PD-L1 expression and VSCC prognosis**	**HR**	**95%CI**	**I^2^**	**Studies**
Overall survival	1.43	1.06 to 1.93	28.9%	7
Progression-free survival	1.57	1.07 to 2.30	38.8%	5
Cancer-specific survival	1.12	0.90 to 1.40	-	1
**PD-L1 expression in intratumoral immune cells and VSCC prognosis**	**HR**	**95%CI**	**I^2^**	**Studies**
Overall survival	1.44	0.59 to 3.51	81.7%	4
Progression-free survival	1.43	0.72 to 2.94	43.6%	3

Abbreviations: CI: confidence interval; CPS: combined positive score; HPV: human papillomavirus; HR: hazard ratio; PD-L1: programmed cell death ligand 1; TC: tumor cell score; VSCC: vulvar squamous cell carcinoma.

## Data Availability

Only publicly available data were used in our study. Data sources and handling of these data are described in the materials and methods section. Further details are available from the corresponding author upon request.

## Supplementary Materials

The following supporting information can be downloaded at: https://www.mdpi.com/article/10.3390/ijms26104594/s1.

## Abbreviations

The following abbreviations are used in this manuscript:

CI	Confidence Interval
CPS	Combined Positive Score
CSS	Cancer-Specific Survival
DFS	Disease-Free Survival
HR	Hazard Ratio
HPV	Human Papillomavirus
IC	Immune Cells
JAK2/STAT1	Janus Kinase 2/Signal Transducer and Activator of Transcription 1
NOS	Newcastle–Ottawa Scale
OR	Odds Ratio
OS	Overall Survival
PD-1	Programmed Cell Death Protein 1
PD-L1	Programmed Death Ligand 1
PI3K/Akt	Phosphoinositide 3-Kinase/Protein Kinase B
PRISMA	Preferred Reporting Items for Systematic Reviews and Meta-Analyses
PROSPERO	International Prospective Register of Systematic Reviews
PFS	Progression-Free Survival
qPCR	Quantitative Polymerase Chain Reaction
Ras/MEK/ERK	Rat Sarcoma/Mitogen-Activated Protein Kinase Kinase/Extracellular Signal-Regulated Kinase
RFS	Recurrence-Free Survival
RR	Relative Risk
TC	Tumor Cells
TCS	Tumor Cell Score
TPS	Tumor Proportion Score
VC	Vulvar Cancer
VSCC	Vulvar Squamous Cell Carcinoma
WHO	World Health Organization
