# Supplementary material for: Positivity Rate of PD-L1 Expression and Its Clinical Significance in Vulvar Cancer: A Systematic Review and Meta-Analysis
Source: Int J Mol Sci. 2025 May 11;26(10):4594. doi: 10.3390/ijms26104594 (PMC12111622; doi:10.3390/ijms26104594)
Supplement: Supplementary file 1 [file ijms-26-04594-s001.zip › ijms-3582696-supplementary.pdf]

Positivity Rate of PD-L1 Expression and Its Clinical Significance in Vulvar Cancer: A Systematic Review and Meta-Analysis

Supplements

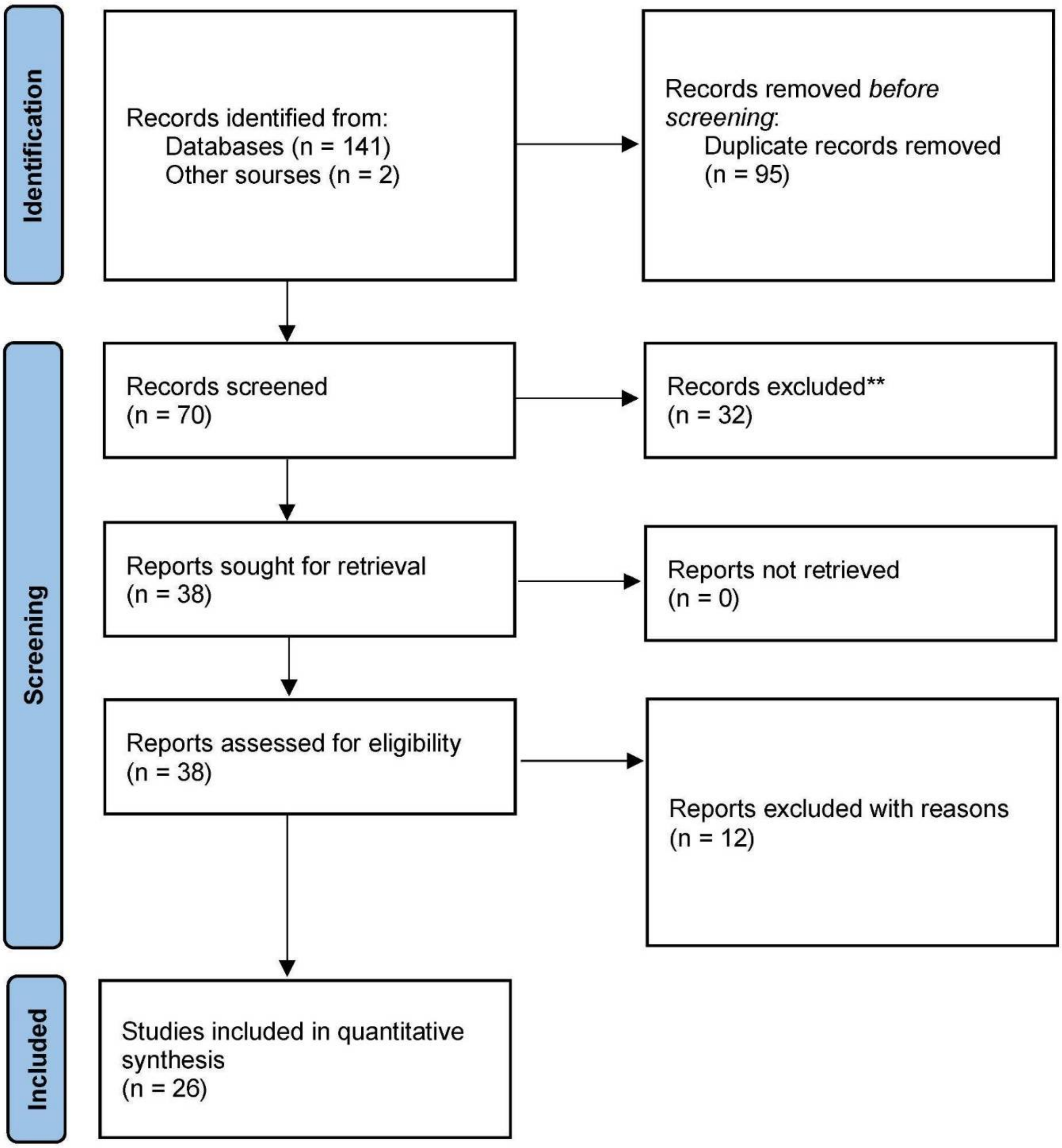

Figure S1. Flow chart of study selection.

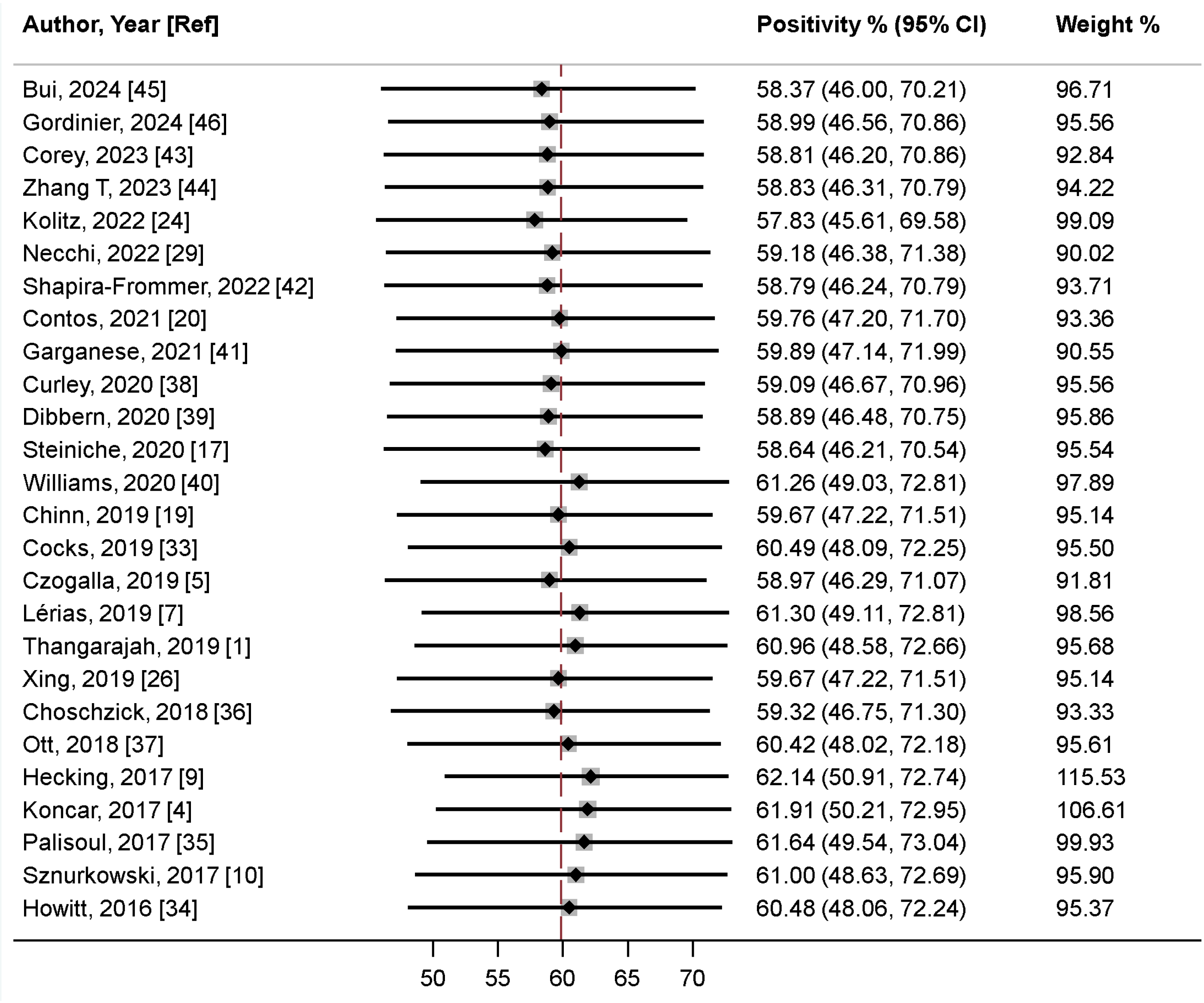

Figure S2. Leave-one-out sensitivity analysis of the summary positivity rate of programmed cell death ligand 1 (PD-L1) expression in vulvar cancer. Each box represents the calculated rate when excluding one study, with the red dashed reference line indicating the original summary positivity rate.

Abbreviations: CI: confidence interval.

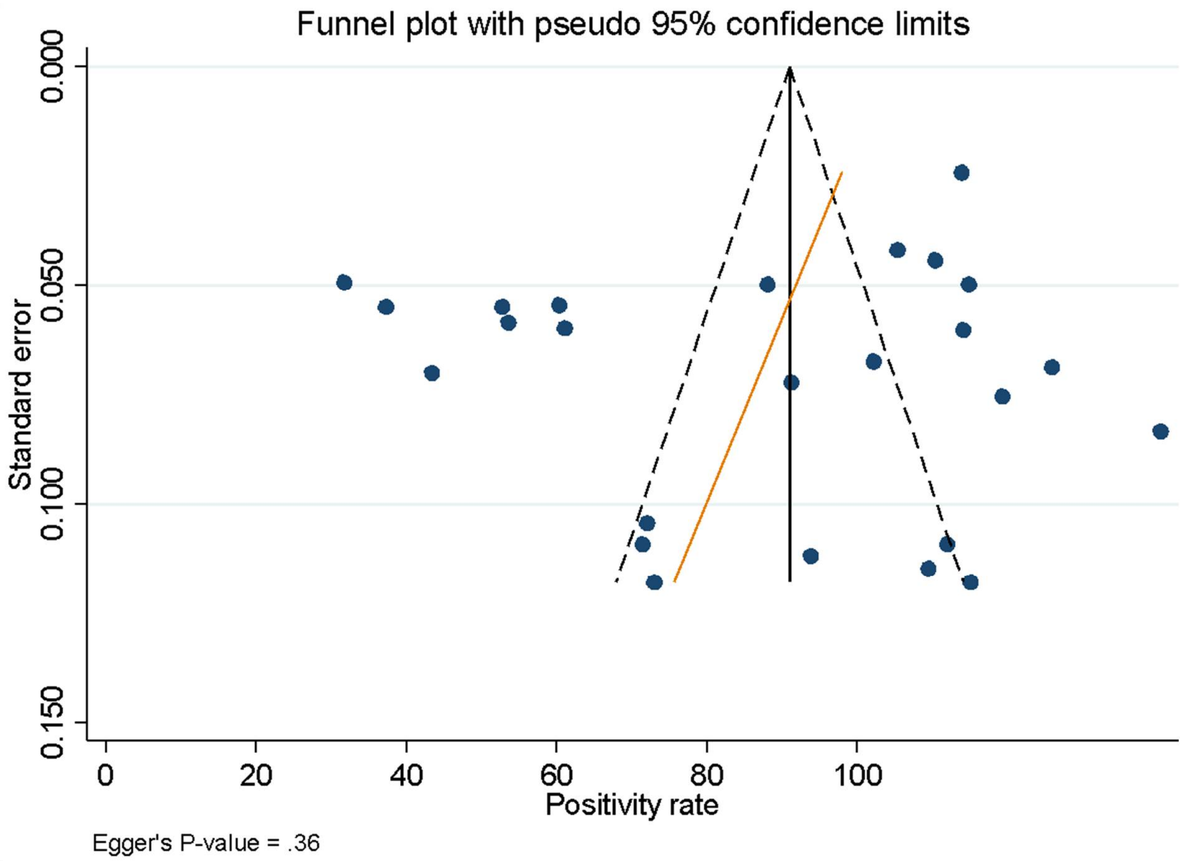

Figure S3. A funnel plot of the summary positivity rate of programmed cell death ligand 1 (PD-L1) expression in vulvar cancer against the standard error, with pseudo 95% confidence limits indicated by the dashed lines. Egger's test P-value = 0.636.

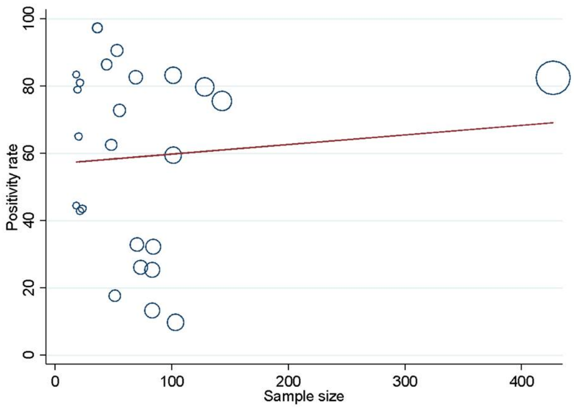

A

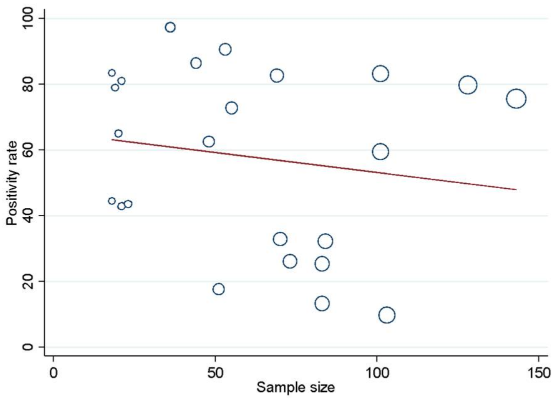

B

Figure S4. Meta-regression of the association between sample size and programmed cell death ligand 1 (PD-L1) expression positivity rate (A) overall, and (B) excluding the one study with the largest sample that could potentially affect the results. No significant association between sample size and positivity rate was observed [(A) p=0.68 and (B) p=0.42].

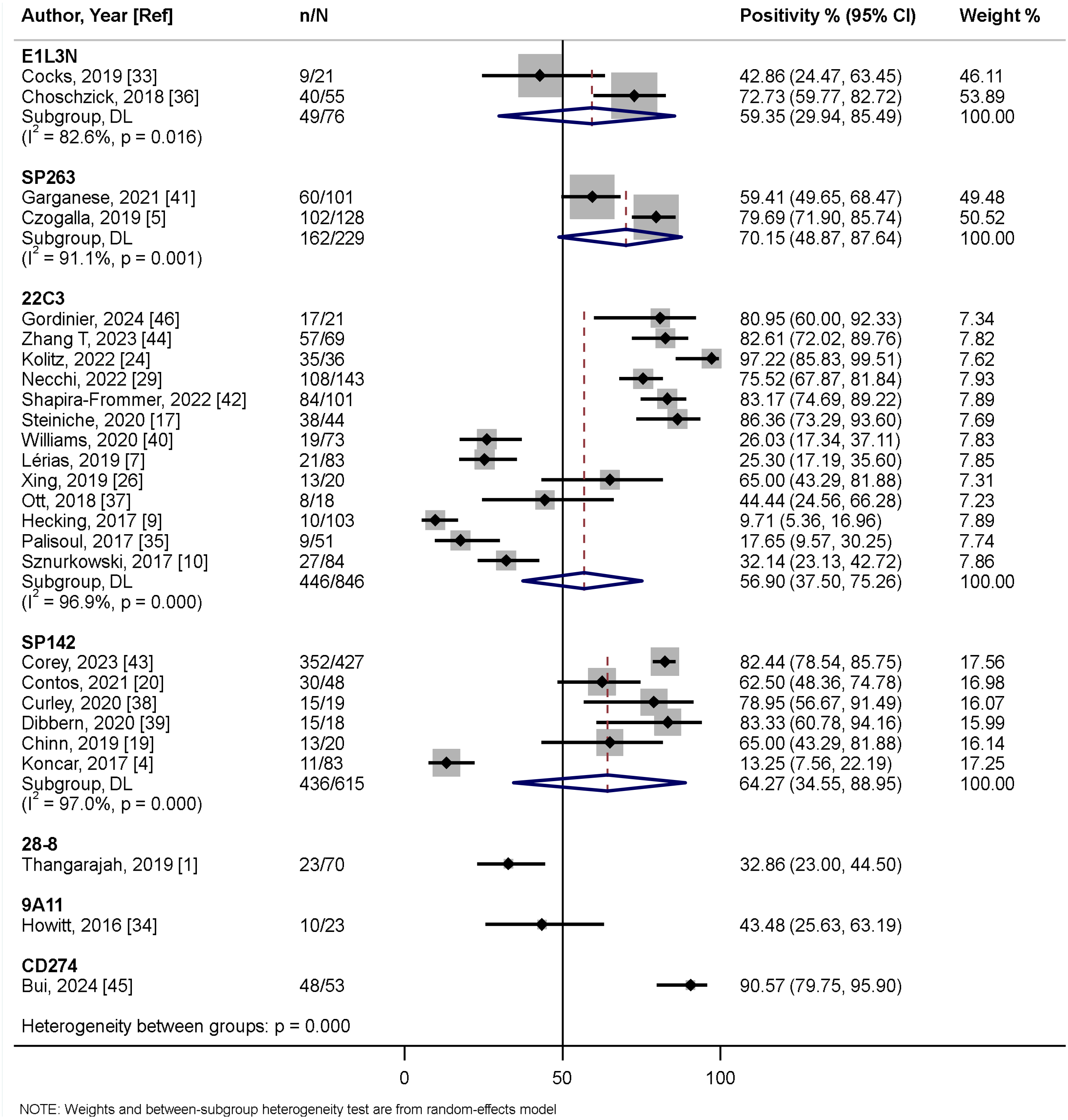

Figure S5. A forest plot of the summary positivity rate of programmed cell death ligand 1 (PD-L1) expression in vulvar cancer, by PD-L1 antibody.

Abbreviations: CI: confidence interval.

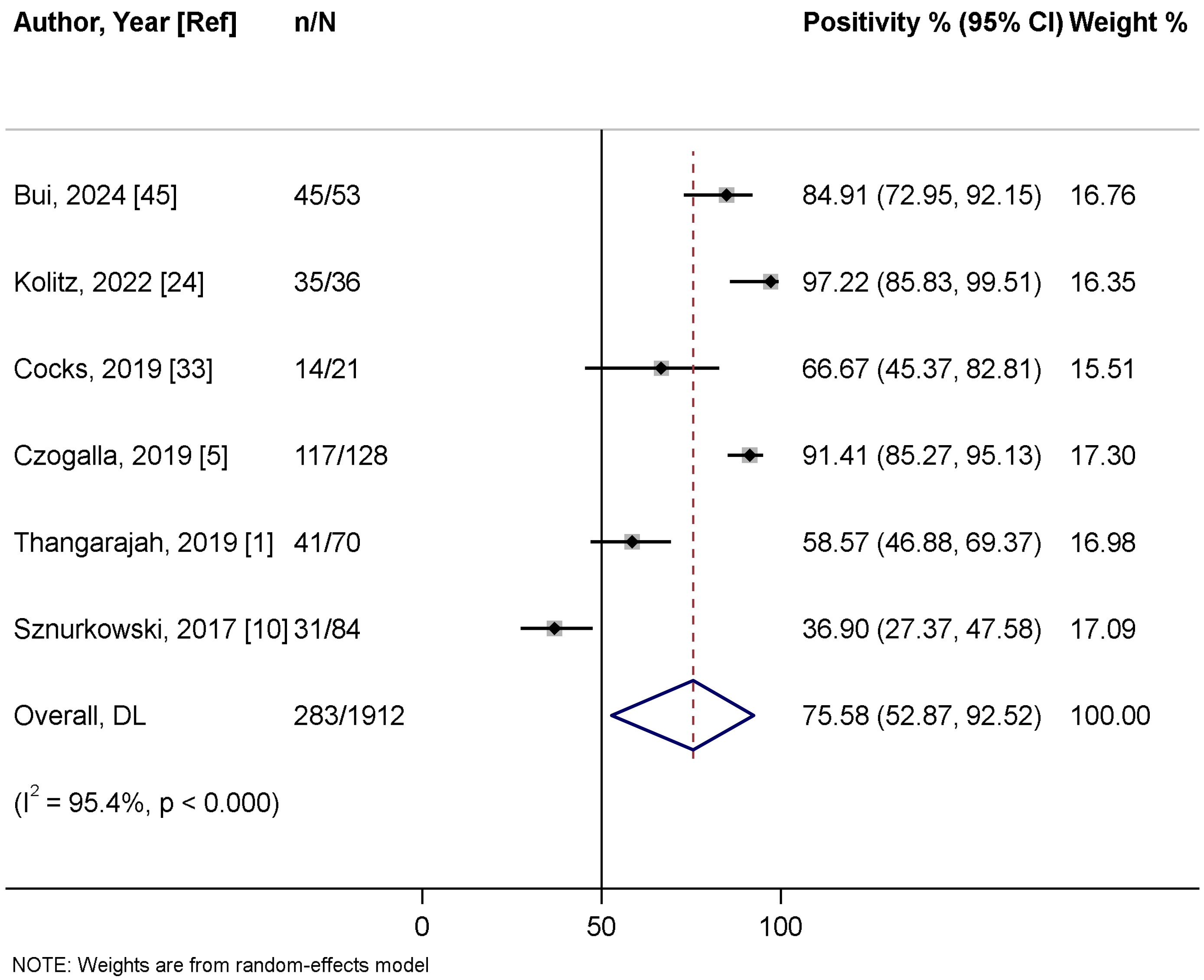

Figure S6. A forest plot of the summary positivity rate of programmed cell death ligand 1 (PD-L1) expression in intratumoral immune cells (ICs) in vulvar cancer.

Abbreviations: CI: confidence interval.

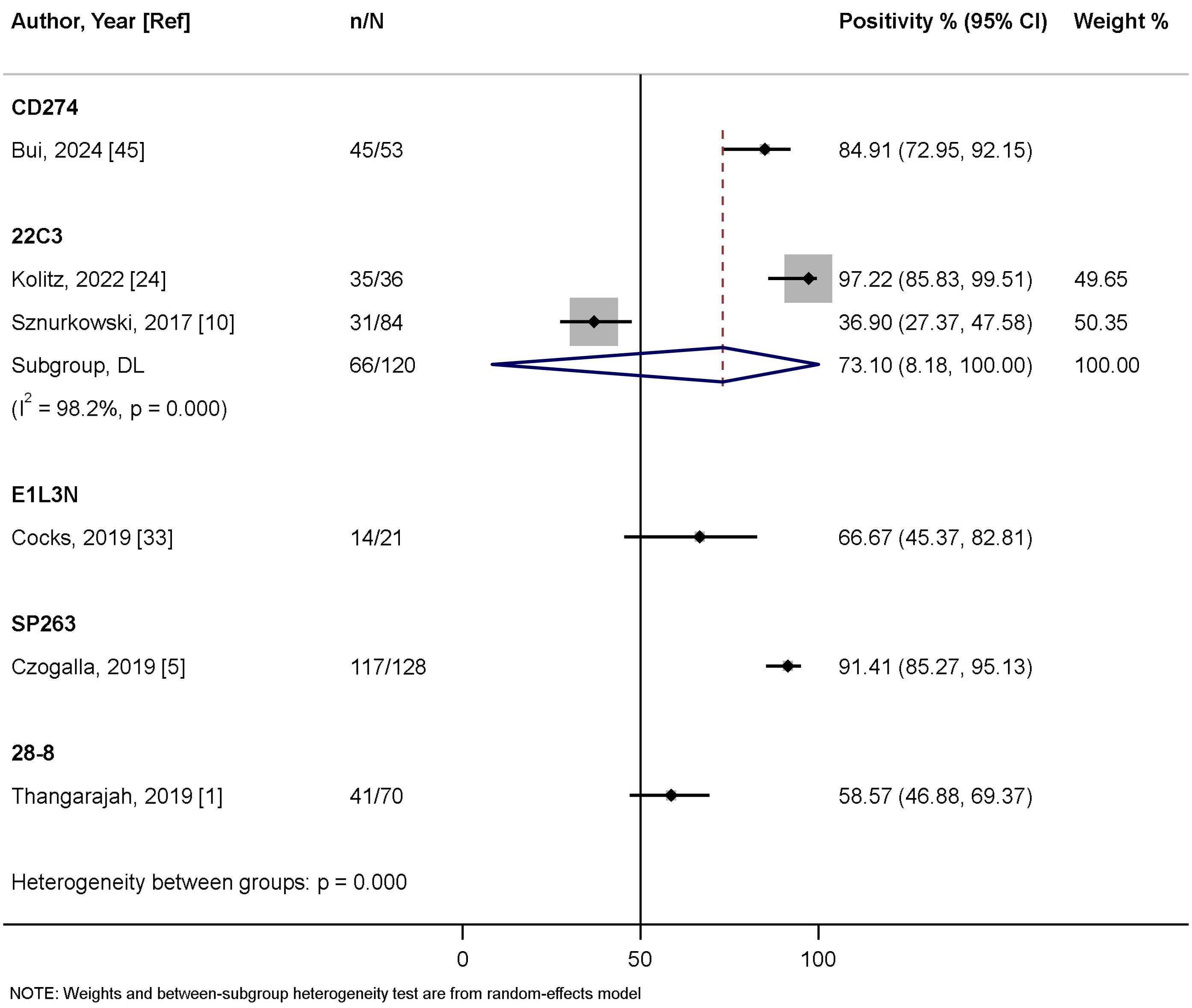

Figure S7. A forest plot of the summary positivity rate of programmed cell death ligand 1 (PD-L1) expression in intratumoral immune cells (IC) in vulvar cancer, by immunohistochemistry antibody.

Abbreviations: CI: confidence interval.

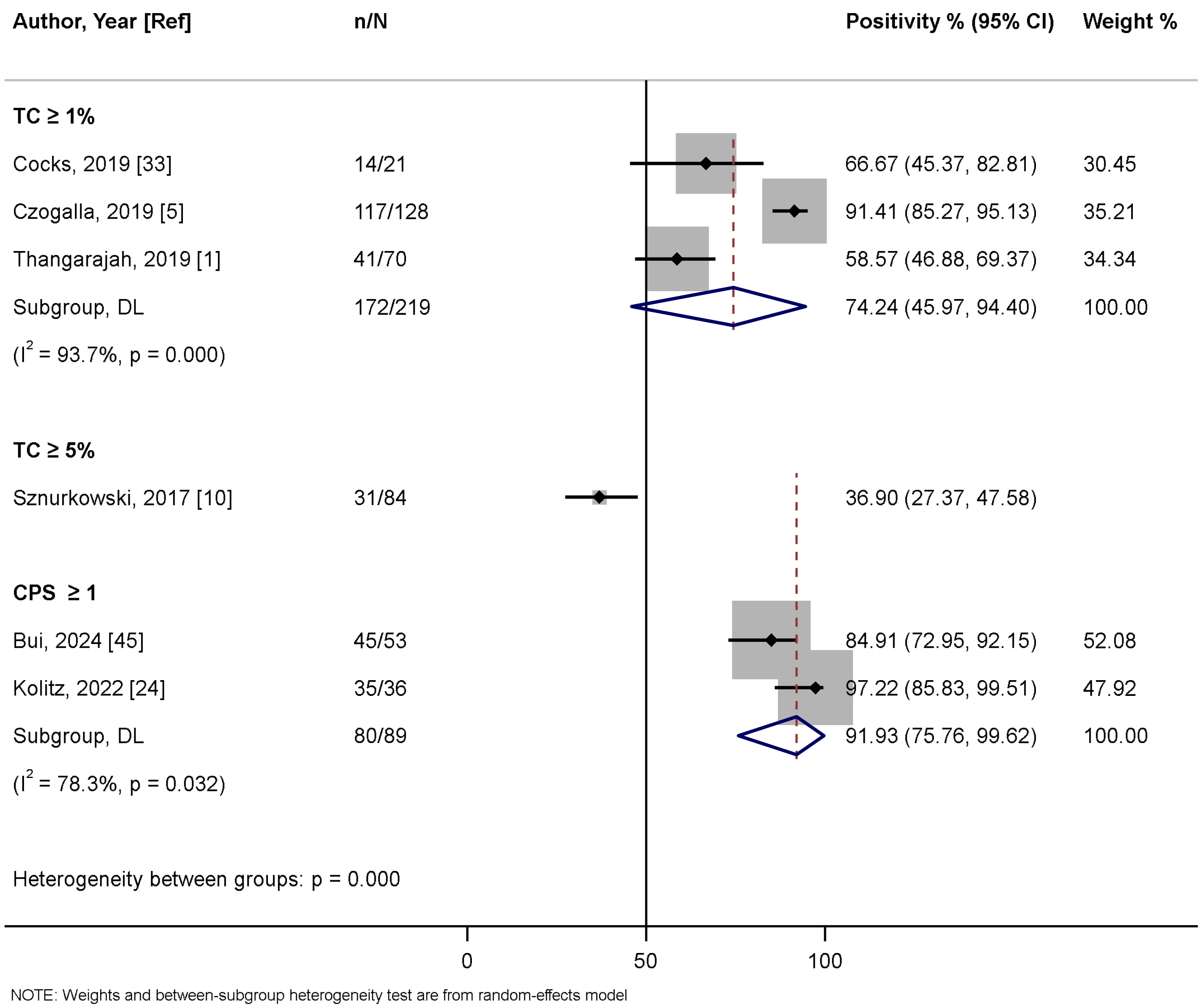

Figure S8. A forest plot of the summary positivity rate of programmed cell death ligand 1 (PD-L1) expression in intratumoral immune cells (IC) in vulvar cancer, by cutoff thresholds used to assess the expression of PD-L1.

Abbreviations: CI: confidence interval.

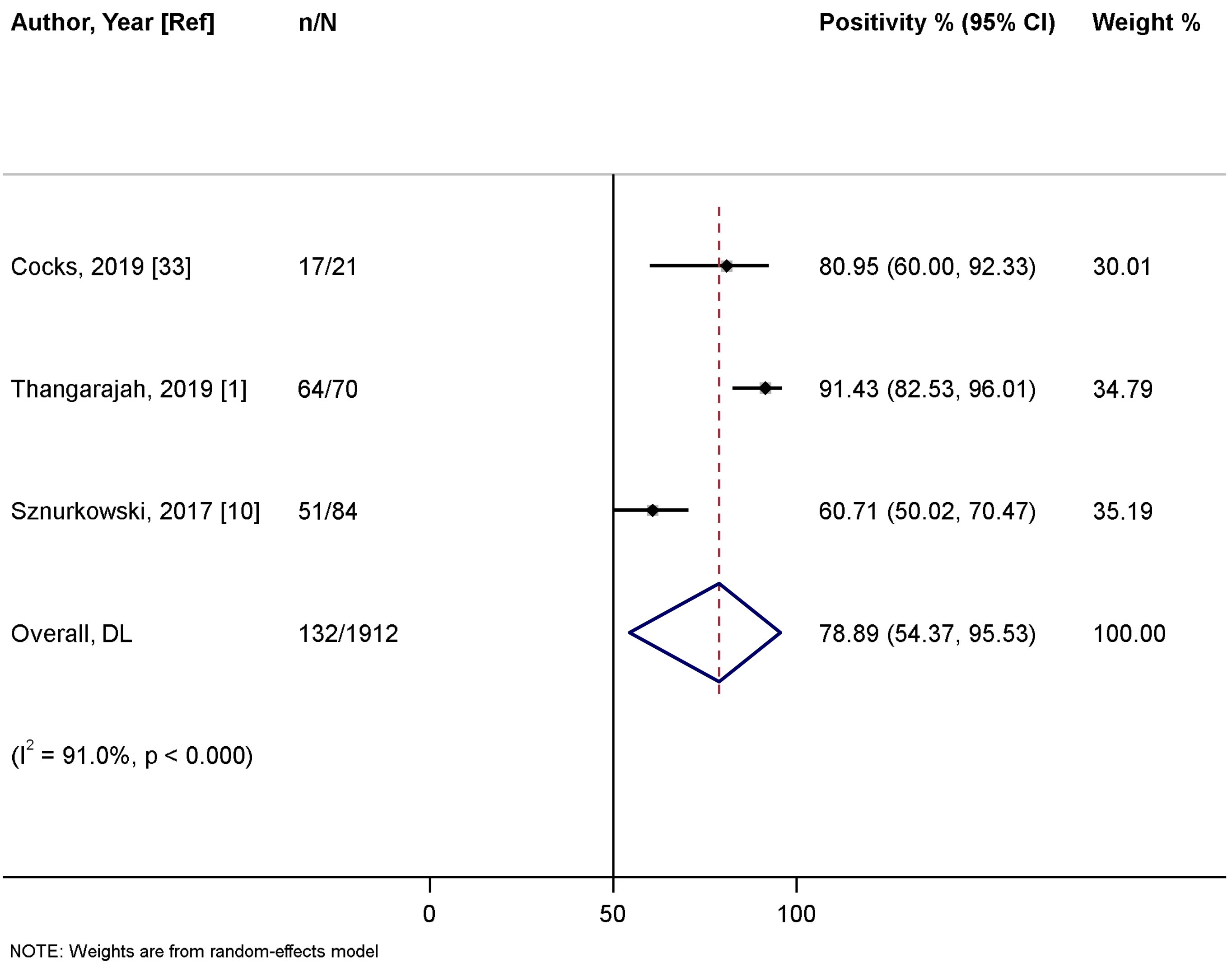

Figure S9. A forest plot of the summary positivity rate of programmed cell death ligand 1 (PD-L1) expression in peritumoral (immune) cells in vulvar cancer.

Abbreviations: CI: confidence interval.

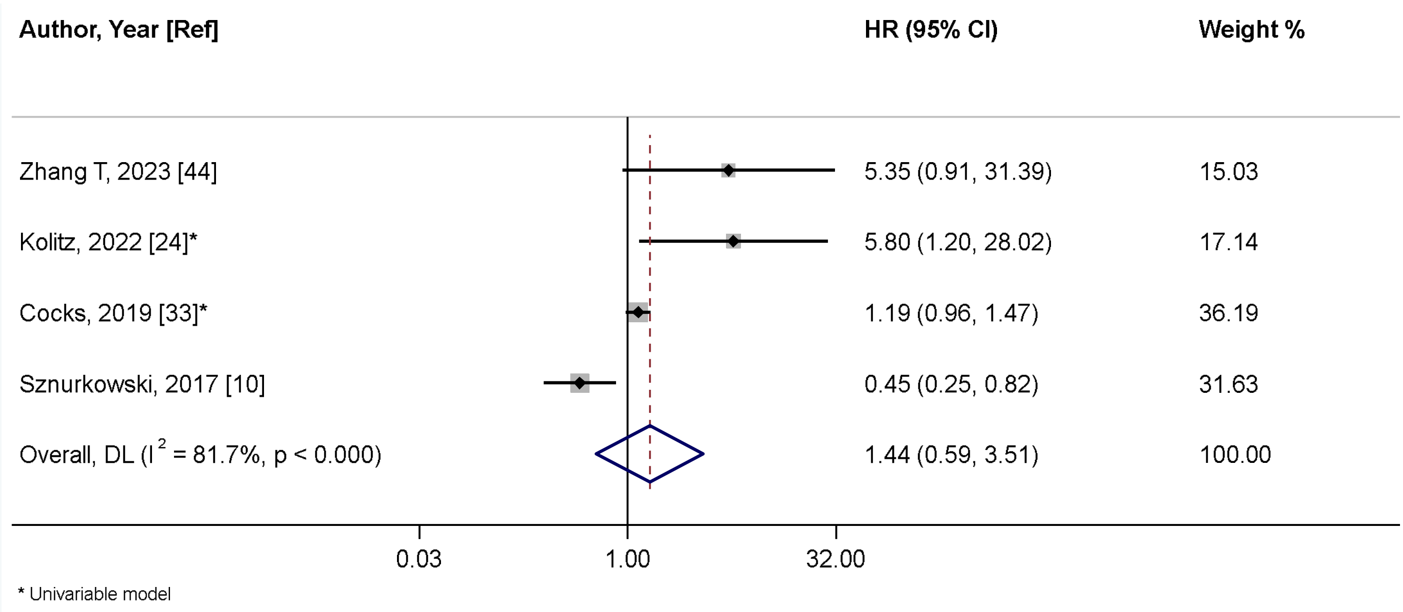

Figure S10. A forest plot of the association between positive versus negative programmed cell death ligand 1 (PD-L1) expression in intratumoral cells and overall survival in vulvar cancer.

Abbreviations: CI: confidence interval; HR: hazard ratio.

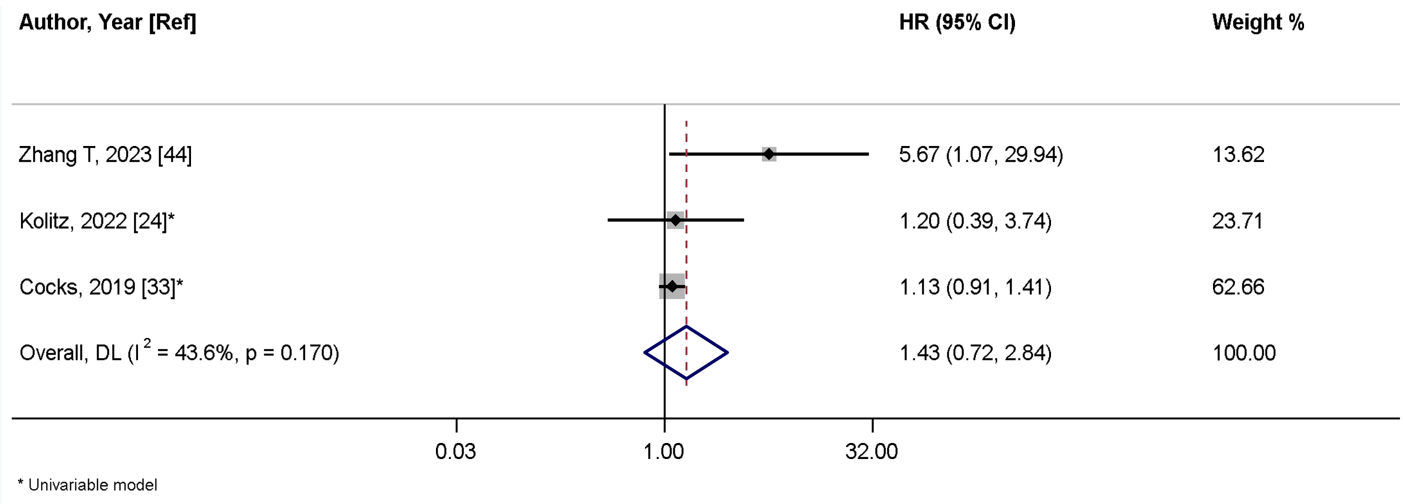

Figure S11. A forest plot of the association between positive versus negative programmed cell death ligand 1 (PD-L1) expression in intratumoral cells and vulvar cancer progression-free survival.

Abbreviations: CI: confidence interval; HR: hazard ratio.

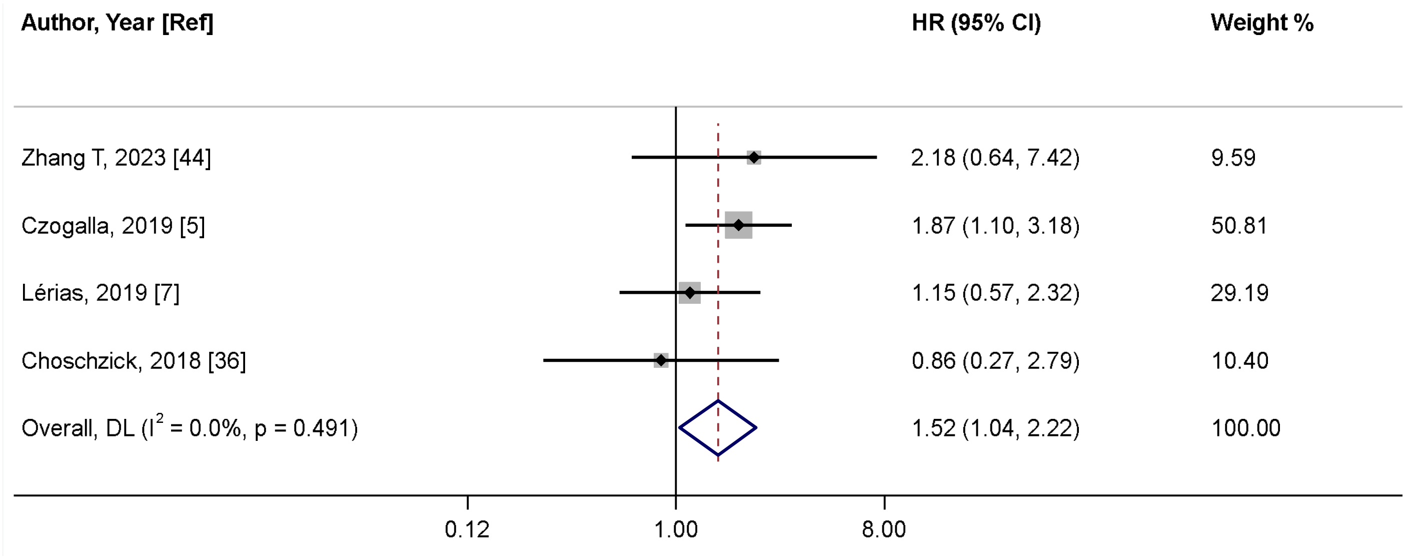

Figure S12. A forest plot of the association between positive versus negative programmed cell death ligand 1 (PD-L1) expression in intratumoral cells and overall survival in vulvar cancer, excluding studies with low study quality.

Abbreviations: CI: confidence interval; HR: hazard ratio.

Table S1. Excluded studies and the reason for exclusion during the full-text screening

**Author, year**	**PMID**	**Reason for exclusion**
Abdulrahman, 2020	32169871	Study in non-cancerous tissues of vulva
Cereceda, 2022	35681797	Number of Cases=1 and no use of Immunohistochemistry
Hou, 2017	28026870	Vulvar melanomas not VSCC
How, 2021	33574401	Case series study
Furlan, 2020	32511773	Non-squamous cell carcinoma of the vulva
Kortekaas, 2021	34716208	RNA expression utilized
Mantovani, 2020	32335720	Review article
Marinelli, 2019	31681606	Review article
Sachdeva, 2021	34234460	Case report
Santoro, 2024	38295614	Review article
Shields, 2018	30016784	Case report and review of the literature study
Winarno, 2022	35291960	Case series study

Table S2. Study quality assessment for each study using the Newcastle–Ottawa Scale (NOS) score.

Author, Year	Selection				Comparability	Outcome			Total score
	Representative sample of relevant population	Selection of the non-exposed cohort	Ascertainment of exposure	Demonstration that outcome of the study does not present at the start of the study	Design of analysis controlled for cofounders	Assessment of outcome	Follow-up adequate for outcomes to occur	Adequacy of follow-up of cohorts	
Hecking, 2017 [9]	★	★	★	★		★	★	★	7
Sznurkowski, 2017 [10]	★	★	★	★	★	★			6
Choschzick, 2018 [36]	★	★	★	★	★★	★			7
Cocks, 2019 [33]	★	★	★	★		★			5
Czogalla, 2019 [5]	★	★	★	★	★★	★	★	★	9
Lérias, 2019 [7]	★	★	★	★	★★	★	★	★	9
Kolitz, 2022 [24]	★	★	★	★		★			5
Zhang, 2023 [44]	★	★	★	★	★★	★	★	★	9

For selection and outcome: *=criterion is satisfied; no *=criterion is not satisfied or cannot be determined.
For comparability: **=criterion is satisfied; *=criterion is partly satisfied; no *=criterion is not satisfied or cannot be determined.
Maximum quality score=9. 0–2 points were considered poor quality; 3-6 points were considered fair quality; 7-9 points were considered as high quality.

Studies adjusted for:
Hecking, 2017 [9]: lymph node involvement
Sznurkowski, 2017 [10]: nodal status, grade, p16
Choschzick, 2018[36]: age, histologic tumor type, tumor stage, nodal stage, grading
Cocks, 2019 [33]: -
Czogalla, 2019 [5]: age, grade, stage
Lérias, 2019 [7]: age, stage
Kolitz, 2022 [24]: -
Zhang, 2023 [44]: age, stage, grade, LMN, HPV, p53, Ki-67, CPS, CD8
